# Vaping-Related Adverse Events and Perceived Health Improvements: A Cross-Sectional Survey among Daily E-Cigarette Users

**DOI:** 10.3390/ijerph18168301

**Published:** 2021-08-05

**Authors:** Melinda Pénzes, Márta Bakacs, Zoltán Brys, József Vitrai, Gergely Tóth, Zombor Berezvai, Róbert Urbán

**Affiliations:** 1 Department of Public Health, Faculty of Medicine, Semmelweis University, H-1085 Budapest, Hungary; 2 National Institute of Pharmacy and Nutrition, H-1051 Budapest, Hungary; Bakacs.Marta@ogyei.gov.hu; 3 Department of Telecommunications and Media Informatics, Faculty of Electrical Engineering and Informatics, Budapest University of Technology and Economics, H-1117 Budapest, Hungary; zoltan.brys@gmail.com; 4 Pharmaproject-Statisztika Ltd., H-2081 Piliscsaba, Hungary; vitrai.jozsef@gmail.com; 5 Institute of Sociology, Centre for Social Sciences, Hungarian Academy of Sciences Centre of Excellence, Eötvös Loránd Research Network, H-1097 Budapest, Hungary; toth.gergely@tk.hu; 6 Faculty of Humanities and Social Sciences, Károli Gáspár University of the Reformed Church in Hungary, H-1091 Budapest, Hungary; 7 Institute of Marketing, Corvinus University of Budapest, H-1093 Budapest, Hungary; zombor.berezvai@uni-corvinus.hu; 8 Institute of Psychology, Eötvös Loránd University, H-1064 Budapest, Hungary; urban.robert@ppk.elte.hu

**Keywords:** e-cigarette, vaping, adverse event, health effect, perceived health, health benefit

## Abstract

Web-based samples of e-cigarette users commonly report significant vaping-related health improvements (HIs) and mild adverse events (AEs). This cross-sectional study with in-person interviewing data collection examined self-reported AEs and perceived HIs among Hungarian adult current daily exclusive e-cigarette (*n* = 65) and dual users (*n* = 127), and former daily e-cigarette users (*n* = 91) in 2018. Logistic regression was used to evaluate associations between reporting any AEs/HIs, vaping status, and covariates. More former users (52.7%) reported AEs than current users (39.6%; *p* = 0.038). Exclusive and dual daily users reported similar rates of AEs (44.6% and 37.0%, respectively; *p* = 0.308). More current users (46.9%) experienced HIs than former users (35.2%; *p* = 0.064). Exclusive daily users were more likely to report HIs than dual users (63.1% versus 38.6%; *p* = 0.001). Former user status and smoking cessation/reduction reasons increased the odds of reporting AEs, whereas nicotine-containing e-liquid use and older age decreased the odds of reporting AEs. Exclusive vaper status, using advanced generation devices, and smoking cessation/reduction reasons increased the odds of experiencing HIs. This study, which used a traditional data collection methodology, found a higher rate of AEs and a lower rate of HIs compared to web-based surveys. Our results highlight that experiencing AEs and HIs is affected by users’ characteristics, in addition to the device and e-liquid type.

## 1. Introduction

The popularity of electronic cigarette (e-cigarette) use is increasing, and many smokers are switching from combustible cigarettes (CCs) to e-cigarettes [1]. Due to the relatively limited number of studies, the rates of adverse events (AEs) or beneficial health impacts of e-cigarettes are not well known. However, proponents of e-cigarettes place more emphasis on the positive aspects of their use, particularly in the public media [2]. E-cigarettes are frequently positioned as a smoking cessation tool or as a clean, easy-to-use, and less harmful device. Moreover, side effects and possible harm associated with their use are rarely mentioned [3,4]. E-cigarettes have been commercially available for more than a decade, and their design and technical characteristics have continuously evolved to provide a more satisfying experience and better nicotine delivery [5,6,7,8]. The growth in the popularity of e-cigarettes among adult tobacco users may be due to the perception that e-cigarettes are an effective smoking cessation aid or harm reduction tool [9,10,11,12]. There is increasing evidence that, overall, vaping is less harmful than smoking CCs. However, vaping is not safe because e-cigarette vapor contains hundreds of potentially toxic and carcinogenic chemicals [13,14]. Recent studies found that the majority of smokers who switched from CCs to e-cigarettes self-reported that vaping has extraordinary beneficial effects on their overall health [12,15,16,17,18]. However, these studies relied predominantly on online samples of e-cigarette users recruited from e-cigarette web shops or forum websites, which might raise concerns regarding the reliability of vapers’ perceived health improvement data due to selection bias [12,15,16,17]. Although an increasing number of studies indicate that smokers who completely switch to e-cigarettes may experience health improvements of the cardiopulmonary and other organ systems, their related biomarkers did not return to the levels of non-smokers [13,19]. Moreover, the long-term impacts of complete switching on morbidity, mortality, and possible health gains remain unclear [13].

Existing studies indicate that smokers switching to e-cigarette use report only mild or moderate AEs over the short to medium term, whereas severe AEs are rarely reported [15,20,21,22]. However, due to the lack of systematic reporting of suspected AEs in many countries, the current knowledge on AEs is mainly based on clinical trials with limited sample sizes [21,23,24], on web-based observational studies with presumably biased samples [15,16,17,22], and, less commonly, on studies using a qualitative interview methodology [10] or data mining from social media and e-cigarette forums [25,26]. Continuous post-marketing monitoring of vaping-related AEs is essential to improve the awareness of consumers, health professionals, and relevant stakeholders regarding the harmful health outcomes of vaping [25].

Of the almost 8.4 million Hungarians 15 years old and older, only 0.6–2.2% were e-cigarette users in the period 2017–2019, and only 0–1.5% were dual users [27,28,29]. Although detailed data on e-cigarette use in Hungary is lacking, a recent review study concluded that e-cigarette use is generally predominant among smokers and young adults in the European population [1]. In Hungary, the relatively low prevalence of current e-cigarette use compared to that in other European countries may be due to a more cautious government position on e-cigarette use and the comprehensive e-cigarette regulatory environment, including bans on e-cigarette use in public places and e-cigarette advertising, and taxation in addition to VAT [27].

Quantitative data collection with in-person interviewing in e-cigarette-related research is becoming increasingly rare due to the dominance of the Internet. Compared to Internet-based surveys, face-to-face interviewing is more expensive, time consuming, and limited to certain geographical areas. However, face-to-face interviewing may have benefits: it can provide higher-quality data due to a better understanding of the questions, an improved completion rate, and greater reliability. Moreover, it may reach less enthusiastic users, and is therefore less prone to response bias [30]. Additionally, compared to open-ended questions, providing a broad, predefined list of vaping-related AEs and possible health changes may improve participants’ willingness to respond and recall their experiences, and reduce memory bias, thus resulting in more accurate data [31]. Compared to non-daily e-cigarette users, it is assumed that daily e-cigarette users have greater exposure to nicotine and toxic compounds by inhaling the e-cigarette vapor; thus, they are more likely to experience possible vaping-related AEs than non-daily e-cigarette users [32]. Furthermore, daily users may also be more likely to experience changes in their health and body functions due to regular vaping compared to non-daily users. We hypothesized that perceived health changes and AEs may have an impact on the continuation or discontinuation of vaping, and on the pattern of use.

This study aimed to assess vaping-related AEs and perceived health improvements among current daily exclusive e-cigarette users (using only an e-cigarette) and daily dual users (using CCs and e-cigarettes alternately), in addition to former daily e-cigarette users who were established cigarette smokers. Additionally, compared to the majority of previous studies, we collected data with in-person interviewing; therefore, we expected a higher rate of AEs and a lower rate of perceived health improvement due to a lower chance of selection bias compared to online surveys. We also sought to explore factors, such as vaping devices and user types (exclusive versus dual), that may have an impact on experiencing AEs and health improvements. Dual users may experience more AEs due to the assumed additive impact of both e-cigarettes and CCs, and they may also report a lower level of health improvement.

## 2. Materials and Methods

### 2.1. Participants and Procedure

A cross-sectional study with tablet-assisted in-person interviewing was conducted by professional interviewers near national tobacco shops and on university campuses in a convenience sample of 2000 persons in Hungary in September–October 2018. The interview was conducted only with individuals who were ≥18 years old, were current or former smokers, and indicated that they had ever tried an e-cigarette. Near national tobacco shops, sex and age-group quota were applied, whereas on university campuses, only students with active university status were interviewed. Consent was obtained from all participants. No financial or other incentive was offered for participation.

Of the initial sample of 2000 current (98.1%) and former (1.9%) smokers, we excluded those who did not confirm that they had ever tried an e-cigarette (*n* = 9). Of the 1991 respondents (recruited near national tobacco shops – not university student: *n* = 991, university student: *n* = 1000), 78.4% had ever tried an e-cigarette (66.4% of not university students versus 90.3% of university students), whereas 20.5% (*n* = 409) used e-cigarettes in the past 30 days (21.7% of not university students versus 19.4% of university students). Almost half of those who had used an e-cigarette in the past 30 days used an e-cigarette on a daily basis (46.9%, *n* = 192), whereas 4.6% (*n* = 91) of the sample were former daily e-cigarette users. Therefore, 283 respondents (not university student: *n* = 162, university student: *n* = 121) were included in the analytical sample of the current study.

### 2.2. Measures

#### 2.2.1. Outcome Variables

Vaping status: Respondents who reported current daily or almost daily e-cigarette use in the past 30 days were regarded as current daily e-cigarette users. Among these, exclusive e-cigarette users were former CC smokers who used only e-cigarettes in the past 30 days, whereas dual users smoked CCs and used e-cigarettes alternately daily or almost daily in the past 30 days. Former daily e-cigarette users were those who had ever used an e-cigarette daily for at least 1 month.

Vaping-related adverse events were assessed among both current and former daily e-cigarette users (“Since you are using/when you have used an e-cigarette, which of the following adverse events have you experienced?”). Based on the commonly reported AEs in the existing literature [15,22], 16 AEs were listed with yes/no response options . Two variables were formed: one was a binary variable that indicated any reported AEs by the respondents, and the other defined the number of reported AEs.

Perceived health improvements were queried among both current daily and former daily e-cigarette users (“Since initiating e-cigarette use/when you have used an e-cigarette, what changes have you experienced in relation to your health, functioning and condition of your body?”) by listing 14 physiological functions [15,22]. Respondents could indicate if they experienced improvement, no change, or worsening in each function. Because perceived health improvements were the focus of the analyses, response options were collapsed into a binary variable (improvement vs. no change/worsening).

#### 2.2.2. Covariates and Control Variables

Vaping behavior: Currently used e-cigarette device type by current e-cigarette users and formerly used device by former e-cigarette users were categorized into older generation (first-generation cigalike and second-generation vape pen), advanced personal vaporizer (APV, third-generation, mechanical or regulated mod), and innovative regulated mod (IRM, extended third/fourth-generation mods with hardware for changing the voltage and/or the output in watts, automatic temperature control, and very low resistance) categories [5,7,33].

Nicotine content of the e-liquid was assessed by predefined responses (nicotine-free, low–cc. 6 mg, medium–cc. 12 mg, high–cc. 18 mg) and, for some analyses, responses were collapsed into a binary variable (nicotine-free vs. nicotine-containing e-liquid).

Participants also indicated their most important reason to initiate vaping from the following response options: (1) to quit smoking or avoid relapsing; (2) to reduce smoking; (3) to use in places where smoking is not allowed; (4) because I thought it would be pleasurable to use an e-cigarette; (5) curiosity/I just wanted to try it; (6) other reason. Response options 3 and 6 were combined due to their low frequencies (4.2% and 3.9%, respectively). Considering the findings of previous studies that indicate smoking cessation and harm reduction are the main reasons for e-cigarette use [11,34], response options were further collapsed into a binary variable (quit smoking/avoid relapsing/reduce smoking vs. curiosity/just to try/pleasurable/other).

Demographic characteristics, such as age, sex, and education, were assessed and used as control variables.

### 2.3. Statistical Analysis

We performed all analyses with IBM SPSS version 25.0. For group comparisons, we conducted chi-square and Mann–Whitney tests, and these analyses were also completed with effect size measures, such as the phi coefficient, Cohen’s d, and odds ratio, wherever applicable. In the case of categorical variables for comparing proportions, we also applied the z-statistic. To predict any AE or any perceived health improvement, we performed multiple binary logistic regression analyses in subsamples of current daily (exclusive versus dual) e-cigarette users and current versus former daily e-cigarette users.

## 3. Results

### 3.1. Characteristics of E-Cigarette User Types

#### 3.1.1. Current Versus Former Daily Users

The comparison of former and current daily e-cigarette users displayed in Table 1 revealed that former daily users are similar to current daily users in many demographic variables; however, former daily users reported the use of first- and second-generation e-cigarettes more frequently than current users. Current users more often vaped with APVs (OR = 2.29; 95% CI = 1.23–4.25) or IRMs (OR = 4.14; 95% CI = 1.80–9.51) compared to former users. These odds ratios remained significant after controlling statistically for age and gender. The most important reason to initiate vaping was to quit smoking or avoid relapsing, both among current and former users. The majority of former daily e-cigarette users returned to daily or non-daily CC use (89.0% and 9.9%, respectively) and only 1.1% successfully quit both smoking and vaping.

#### 3.1.2. Dual Users Versus Exclusive E-Cigarette Users

Exclusive e-cigarette users tended to be younger than dual users but did not differ significantly in other demographic characteristics (Table 1). Exclusive e-cigarette users were more likely to have vaped with IRMs (OR = 4.68; 95% CI = 2.11–10.40) compared to dual users; however, there was no significant difference in the case of APV use (OR = 1.41; 95% CI = 0.66–3.01). The majority of exclusive e-cigarette users initiated vaping to quit smoking or avoid relapsing, whereas dual use was more related to reduce smoking.

### 3.2. Adverse Events of E-Cigarette Use

Almost 40% of current daily e-cigarette users reported experiencing any AE (39.6% (32.6–46.9%)); this proportion was higher among former daily users (52.7% (42.0–63.3%)) and they were significantly more likely to report AEs than current daily users (OR = 1.70; 95% CI = 1.03–2.82) (Table 2). The mean number of self-reported AEs was also significantly higher among former users than current users. Regarding current user types, slightly more exclusive users reported experiencing any AE than dual users, but the difference was not significant (OR = 1.37; 95% CI = 0.75–2.52).

Frequently reported AEs—by 10% or more of current users—were (1) sore/dry mouth and throat; (2) cough; (3) burning, scratchy feeling in the mouth, lips, and throat; and (4) headache. Former users also frequently reported additional AEs, such as (5) palpitation; (6) breathing difficulties; (7) dizziness; (8) weakened taste; and (9) sleepiness. Exclusive e-cigarette and dual users did not differ significantly in the proportions of these nine AEs, with the exception of weakened taste, which was more frequently reported by dual users. Several less frequently mentioned AEs were significantly more likely to be reported among former daily users, such as allergy, painful, swollen and red tongue or mouth, and insomnia, compared to current daily users. Almost half of the listed AEs were not experienced by exclusive users at all, although dual users reported these with low frequency. Binary logistic regression analyses were conducted to explore the association between nicotine-containing or nicotine-free e-liquid use and experience of AEs (Table 3). With the exception of palpitation, AEs overall were less likely to occur among those who used nicotine-containing e-liquid, although this association was significant only in the cases of dizziness, weakened taste, gingivitis and gum bleeding, chest pain, and black tongue.

Reporting of any AE was not related to demographic variables, user types, or other covariates among current daily users in a multivariate analysis (Table 4). It is important to note, however, that in univariate analysis, users of IRM e-cigarettes were more likely to report AEs than users of cigalike or vape pen devices (OR = 2.72; 95% CI = 1.47–5.04), although this effect was absent in the multiple logistic regression analysis. Regarding current versus former user groups, reporting any AE was less likely among participants older than 30 years and those who used nicotine-containing e-liquid, whereas former users and those who initiated vaping with reasons such as smoking cessation or smoking reduction were more likely to have experienced any AE (Table 5). Contrary to the current user group, in univariate analysis, former users of APVs or IRMs did not experience any AEs with significantly different odds than former users of cigalike or vape pen devices (OR = 0.62; 95% CI = 0.22–1.76; OR = 2.77; 95% CI = 0.51–15.06, respectively).

### 3.3. Perceived Health Improvement Due to E-Cigarette Use

As shown in Table 2, more than 40% of current daily e-cigarette users reported at least one health-related improvement due to e-cigarette use (46.9% (39.7–54.2%)), and this proportion was non-significantly lower for former daily users (35.2% (25.4–45.9%), OR = 0.62; 95% CI = 0.37–1.03). Among current daily users, almost two-thirds of exclusive e-cigarette users reported at least one perceived health improvement with almost three-fold higher odds than dual users (OR = 2.72; 95% CI = 1.47–5.04). The mean number of perceived health improvements was significantly higher among both current and exclusive users than among former and dual users, respectively.

The most frequently mentioned perceived health improvements were related to breathing, morning cough, taste, smell, endurance, physical status in general, and mood. With the exceptions of physical status in general and endurance, perceived health benefits were experienced with significantly lower proportions by former daily users compared to current users. We also observed significant differences between exclusive e-cigarette users and dual users. Exclusive e-cigarette users were more likely to have reported an improvement in overall physical status (OR = 3.33; 95% CI = 1.65–6.71); breathing (OR = 3.12; 95% CI = 1.67–5.85); stress tolerance (OR = 2.64; 95% CI = 1.22–5.72); endurance (OR = 2.61; 95% CI = 1.32–5.19); quality of sleep (OR = 2.40; 95% CI = 1.02–5.61); taste (OR = 2.36; 95% CI = 1.23–4.51); mood (OR = 2.24; 95% CI = 1.12–4.54); and smell (OR = 2.17; 95% CI = 1.12–4.19) than dual users.

In multiple logistic regression analysis, exclusive e-cigarette users, users of IRMs, and those whose reason for initiating vaping was smoking cessation or smoking reduction were significantly more likely to have experienced any perceived health improvement (Table 4). A similar association was found in the current and former daily user groups; however, the user type (former or current) was not related to reporting any perceived health improvement (Table 5).

## 4. Discussion

In our study, almost 40% of current daily e-cigarette users and more than 50% of former daily e-cigarette users experienced any AE since they had initiated vaping. Our findings indicate that AEs are relatively common among e-cigarette users. In line with previous studies, mouth and throat dryness and irritation, cough, and headache were the most frequently reported AEs, particularly in our current daily e-cigarette user sample [15,22,23,24,25,35,36,37]. The rate of AEs varies across studies with different study designs between 7–58% [15,22,24,35,36,38,39,40]. In a meta-analysis by Liu et al. (2018), reported AE rates were found to be higher in web-based surveys than in clinical trials [36], although some more recent online questionnaire-based studies showed remarkably lower rates [15,35]. According to these, the proportion of reporting any AE and some specific AEs explored in our quantitative study with an in-person interviewing method is consistent with the higher range of AE rates. Higher reported AEs rates are perhaps due to the less biased sampling method. Furthermore, we also used a more detailed list of AEs, which could prompt a higher rate of recalling events. An additional reason for higher AE rates could be that more than two-thirds of our sample had a higher educational level. Other studies with highly educated samples also tend to report AEs with greater frequency [22,24,40]. Other sample characteristics, including the mean age, sex, the most commonly used device type, and nicotine concentration of the e-liquid, did not differ markedly from previous studies [15,22,24,37,38,40].

Our results suggest that e-cigarette users can experience AEs irrespective of the vaping pattern (exclusive or dual use), although exclusive users who used the most recent generation of e-cigarettes indicated that they experienced slightly more AEs overall. An explanation for this association may originate from the device characteristics. E-cigarette aerosol composition is affected by engineering, individual modifications, and the design features of the device. Fourth-generation Innovative Regulated Mods are more powerful than older generation e-cigarettes, and they can generate and release a greater amount of aerosol and toxicants [14,33]. Moreover, improper maintenance of devices, e.g., ignoring the recommended timing of coil replacement or e-cigarettes with lower-quality metal components, can also increase toxicant emissions [14,41]. Furthermore, flavored e-liquids can generate a large quantity of new chemicals during aerosolization [14,42]. In combination, these device and e-liquid characteristics may cause increased AE perceptions by users.

It appears that there are differences between exclusive and dual daily e-cigarette users in experiencing specific AEs, although the statistical power to detect the difference may be low due to the relatively small sample size. Dual users indicated several AEs (e.g., weakened taste; chest pain; black tongue; and painful, swollen, and red tongue or mouth) that were not experienced by any exclusive vapers. Our results suggest that the majority of listed AEs were unrelated to nicotine. It is speculated that oral AEs may be due to other inhaled chemicals from the e-liquid, changes in intraoral pH, mucosal drying, or altered oral immune response [43]. Other AEs may be associated with inhaled e-liquid irritants, puffing characteristics, and related changes in breathing [44]. Nevertheless, the extent to which the dual use itself generated these AEs is unclear. Contrary to previous studies that found that dual users experienced more AEs, our study did not explore significant differences in reporting AEs among this group compared to exclusive users [24,45,46].

In our study, former daily e-cigarette users reported almost two-fold higher odds of experiencing any AE than current daily users, which may indicate less commitment bias towards the positive expectancies of vaping. Our results suggest that young adult former daily e-cigarette users who used nicotine-free e-liquid, and initiated vaping to quit smoking or to reduce smoking, experienced more AEs during their previous daily vaper experiences. Young adult smokers who start vaping may be more health conscious and have greater expectancies regarding the health benefits of e-cigarette use than older individuals with decades of smoking history. In addition to health benefit issues, recreational reasons may also be a driver for younger adults to initiate vaping with attractive and “high-tech” advanced generation e-cigarettes and flavored, nicotine-free e-liquids, which they perceive to be less harmful than earlier generation devices and nicotine-containing e-liquids [37,46]. However, switching from smoking to vaping, and drastically decreasing the nicotine intake, may increase the perception of AEs in several ways. It is assumed that by using nicotine-free e-liquid, the reinforcing effect of nicotine may be missing; therefore, perceptions of AEs may be enhanced. Furthermore, nicotine-free e-liquids may include ingredients that differ in concentration or are not used in nicotine-containing e-liquids, and that can intensify the occurrence of AEs. Increased perception of AEs could be a reason for discontinuing e-cigarette use in addition to other dissatisfying characteristics of vaping, including poor craving alleviation and negative sensory experiences [9,47,48]. Improper selection of the e-cigarette device, the e-liquid flavor, and nicotine concentration may also increase the occurrence of AEs. Previous studies found that more advanced generation devices provide nicotine delivery similar to that of CCs [8,49]. Such advanced generation e-cigarettes combined with higher nicotine content e-liquids and excessive use of e-cigarettes may explain some of the AEs (e.g., palpitation, headache, dizziness, and chest pain) experienced with greater likelihood by former daily users [13,37,50]. Other AEs (e.g., respiratory and oral irritation, and allergy) are supposed to be flavor specific because numerous flavoring chemicals have a respiratory irritant effect [13,35,49]. However, it is unclear whether these previous AEs contributed to quitting e-cigarettes and switching back to CC use, or if the recall of AEs was distorted by memory bias. Further prospective research is required to clarify if AEs motivate the decision to quit e-cigarettes.

This study found that almost half of current daily e-cigarette users experienced any health improvement, and exclusive daily users reported higher rates (63%) than dual users (39%). Our results are consistent with other studies indicating that dual users perceived less improvements to their health than exclusive e-cigarettes users [11,15,17,22,46]. The reason for poorer perceived positive health outcomes could be that dual use does not result in substantial harm reduction compared to a complete switch from smoking to vaping [13,51]. Thus, potential health improvements due to vaping are significantly less among dual users than among exclusive e-cigarette users. Previous studies detected remarkable perceived health improvements among e-cigarette users; however, in our sample, exclusive vapers reported moderate improvements, and dual users reported only modest health improvements [12,15,16,18,22]. It is particularly striking that in a recent online questionnaire-based study conducted among visitors of Hungarian e-cigarette forums and a web shop, participants perceived improvements in their body function in much higher rates (28–90% for specific physiological functions) than in our current study [15]. Because our sample did not differ markedly from this and other previous studies regarding some sample characteristics (e.g., mean age, sex, educational level, the most commonly used device type, and nicotine concentration of the e-liquid) [15,17,18,22], an explanation for the difference could be that the sampling methodology may significantly distort the perceived health-related outcomes of e-cigarette use. It is assumed that questionnaire-based surveys with in-person interviews may be less prone to selection bias towards more enthusiastic e-cigarette users with disproportionately more positive perceptions and experiences of vaping than online surveys, particularly those posted on vaping-related websites [36].

Similar to other studies, respiratory, taste, smell, and overall physical improvements were experienced most frequently by our sample [12,15,16,17,22,37,46]. However, the rates of these perceived health improvements were substantially lower than in previous potentially biased samples [15,16,17,22]. In addition to improvements in physical functioning, a small number of studies explored the mental health benefits of vaping [12,15,16,22,46]. Our results also confirmed significant mental health improvements, including improved mood, stress tolerance, and sleep quality, particularly among exclusive e-cigarette users.

Although current daily e-cigarette users reported improvements in almost all listed physiological changes with higher rates than former users, differences were non-significant. This suggests that some negative aspects of vaping may outweigh the experienced health benefits and can encourage users for discontinuation [37,48]. Exclusive IRM type e-cigarette users aiming to quit or reduce smoking experienced health improvements with greater likelihood than users of earlier generation devices, those whose reason for initiating vaping was other than cessation, and dual users. These results highlight that, for many smokers, switching from smoking to vaping by itself may be not enough to experience possible health benefits. Therefore, managing personalized device choice by considering reasons for use and enhancing quitting behavior could result in better compliance with e-cigarette use. Nevertheless, it should be noted that some professional organizations recommend the short-term use of nicotine-containing e-cigarettes as a smoking cessation aid for those smokers who are unable to quit with first-line cessation medications [13,52,53]. However, the ultimate goal should be the complete cessation of vaping to eliminate the residual health risks of vaping [52,53].

The strengths of our study include the in-person interviewing data collection methodology, which may be less prone to selection bias. In addition, measuring health changes and AEs using a detailed list of AEs and physiological conditions constructed according to previous research findings may facilitate participants’ recall of their health changes. Our results confirm that applying different sampling methods could be a source of variation in the results, even if the questions are identical [15,54]. However, our findings should be interpreted with caution due to several limitations. The self-reporting may be prone to bias due to social desirability. An extensive experience sampling methodology from the beginning of use would be helpful to clarify both AEs and health improvements; however, performing this kind of study has ethical barriers. Cross-sectional data do not allow the formation of any conclusions regarding causality; for example, we cannot be certain that former users quit vaping because of perceived AEs or for other reasons. Furthermore, the present study was based on a convenience sample; therefore the generalization of our results is also limited. An additional limitation could be that, although predefined lists of vaping-related AEs and possible health changes may improve participants’ recall and reduce memory bias, predefined lists may cue participants to respond according to the researchers’ expectations [31]. Our results exploring the predictors of AEs and perceived health improvements due to e-cigarette use should be interpreted with caution due to the relatively large quantity of missing data in multiple regression models. Our sample size also did not allow detection of the less frequent AEs, such as black tongue. The surveillance of AEs in large samples would help to identify the rare but serious adverse events. Furthermore, the large variability of devices makes it difficult to evaluate AEs. Finally, our respondents reported AEs that they had experienced since they had started to use e-cigarettes; therefore, we were not able to detect if these AEs declined over time.

## 5. Conclusions

Although the majority of AEs were mild in our sample, the relatively high proportion of reported AEs confirms that e-cigarette use is not without health risks. It is necessary to improve the safety of e-cigarette devices and e-liquids, and to reduce the likelihood of adverse health consequences of vaping [14,37]. Therefore, stricter regulations for manufacturers, and both mandatory—for manufacturers, importers, and distributors—and voluntary—for users and health professionals—systematic reporting of suspected AEs, in addition to continuous surveillance of reported AEs, by national competent authorities is essential [14,25,55]. Furthermore, it is important to improve consumers’ awareness that e-cigarettes are not a benign alternative to conventional tobacco products, because their use is commonly associated with negative health outcomes [25]. Our results indicated that, although a wide range of perceived health improvements was experienced, particularly by exclusive vapers, the proportion of daily e-cigarette users who did experience these improvements was significantly below the shares found in web-based surveys. Considering traditional data collection methods in e-cigarette research, these may provide additional knowledge to better understand e-cigarette users’ characteristics, and their experiences of health benefits and health harms. Finally, it is essential to replace the largely anecdotal experiences of healthcare professionals on the safety and efficacy of e-cigarettes, and provide evidence-based educational materials to enable them to guide their patients to make an informed decision regarding the use of e-cigarettes [52].

## Figures and Tables

**Table 1:** Descriptive characteristics of current daily and former daily e-cigarette users.

Variable	Ever Daily E-Cigarettes Users, *n* = 283				Current Daily E-Cigarette Users, *n* = 192			
	Current Daily E-Cigarette Users *n* (%) 192 (67.8)	Former Daily E-Cigarette Users *n* (%) 91 (32.2%)	*p*-Value ^a^	ES	Exclusive E-Cigarette Users *n* (%) 65 (33.9)	Dual Users *n* (%) 127 (66.1)	*p*-Value ^b^	ES
**Sex**			0.197	φ = 0.08				
Male	133 (69.3)a	56 (61.5)a			48 (73.8)a	85 (66.9)a	0.326	φ = 0.07
Female	59 (30.7)a	35 (38.5)a			17 (26.2)a	42 (33.1)a		
**Age (Years)**								
Mean (SD)	33.3 (15.7)	35.6 (17.3)	0.394	d = 0.14	29.2 (12.6)	35.4 (16.7)	0.067	d = 0.42
18–30 years	119 (62.0)a	50 (54.9)a	0.455	φ = 0.08	48 (73.8)a	71 (55.9)b	0.053	φ = 0.18
31–59 years	55 (28.6)a	29 (31.9)a			13 (20.0)a	42 (33.1)a		
60+ years	18 (9.4)a	12 (13.2)a			4 (6.2)a	14 (11.0)a		
**Education**								
Technical school or less	60 (31.4)a	40 (44.0)b	0.120	φ = 0.12	15 (23.1)a	45 (35.7)a	0.101	φ = 0.16
High school or vocational school	115 (60.2)a	45 (49.5)a			46 (70.8)a	69 (54.8)b		
College or university	16 (8.4)a	6 (6.6)a			4 (6.2)a	12 (9.5)a		
**Nicotine Concentration of the E-Liquid**			0.516	φ = 0.10			0.904	φ = 0.06
nicotine-free (0 mg)	20 (11.8)a	13 (16.0)a			7 (12.3)a	13 (11.6)a		
low (~6 mg)	114 (67.5)a	47 (58.0)a			37 (64.9)a	77 (68.8)a		
medium (~12 mg)	28 (16.6.)a	16 (19.8)a			11 (19.3)a	17 (15.2)a		
strong (~18 mg)	7 (4.1)a	5 (6.2)a			2 (3.5)a	5 (4.5)a		
**E-Cigarette Device**			< 0.001	φ = 0.24			<0.001	φ = 0.30
Cigalike or vape pen	71 (38.8)a	50 (64.1)b			17 (26.2)a	54 (45.8)b		
Advanced Personal Vaporizer	65 (35.5)a	20 (25.6)a			20 (30.8)a	45 (38.1)a		
Innovative Regulated Mod	47 (25.7)a	8 (10.3)b			28 (43.1)a	19 (16.1)b		
**Reasons to Use an E-Cigarette**			0.111	φ = 0.16			<0.001	φ = 0.46
to quit smoking or avoid relapsing	72 (37.5)a	28 (30.8)a			44 (67.7)a	28 (22.0)b		
to reduce smoking	56 (29.2)a	27 (29.7)a			6 (9.2)a	50 (39.4)b		
pleasurable to use an e-cigarette	27 (14.1)a	12 (13.2)a			7 (10.8)a	20 (15.7)a		
curiosity/just wanted to try	19 (9.9)a	19 (20.9)b			5 (7.7)a	14 (11.0)a		
other reason	18 (9.4)a	5 (5.5)a			3 (4.6)a	15 (11.8)a		

Note: ^a^ Chi-square test or Mann–Whitney test *p*-values were used to examine the differences between current daily and former daily e-cigarette users. ^b^ Chi-square test or Mann–Whitney test *p*-values were used to examine the differences between exclusive e-cigarette users and dual users. ES: effect size. d: Cohen’s d. φ: phi coefficient. Different letters indicate significant difference (at *p* < 0.05) in proportion according to the z-test.

**Table 2:** Prevalence of vaping-related adverse events and perceived improvements in physiological functions among current daily e-cigarette users and former daily e-cigarette users.

	Ever Daily Users, *n* = 283				Current Daily EC Users, *n* = 192			
Variable	Current Daily EC Users *n* (%) 192 (67.8)	Former Daily EC Users *n* (%) 91 (32.2)	*p* ^a^	Odds Ratio ^c^/ Cohen’s d	Exclusive EC Users *n* (%) 65 (33.9)	Dual Users *n* (%) 127 (66.1)	*p* ^b^	Odds Ratio ^d^/ Cohen’s d
**Adverse Events**								
Sore/dry mouth and throat	42 (21.9)	23 (25.3)	0.525	1.21	18 (27.7)	24 (18.9)	0.163	1.64
Cough	37 (19.3)	24 (26.4)	0.175	1.50	10 (15.4)	27 (21.3)	0.329	0.67
Burning, scratchy feeling in the mouth, lips, and throat	23 (12.0)	24 (26.4)	**0.002**	**2.63**	7 (10.8)	16 (12.6)	0.712	0.84
Headache	19 (9.9)	15 (16.5)	0.111	1.80	4 (6.2)	15 (11.8)	0.214	0.49
Palpitation	15 (7.8)	15 (16.5)	**0.027**	**2.33**	8 (12.3)	7 (5.5)	0.097	2.41
Dizziness	12 (6.3)	13 (14.3)	**0.026**	**2.50**	3 (4.6)	9 (7.1)	0.373	0.63
Breathing difficulties	11 (5.7)	15 (16.5)	**0.003**	**3.25**	5 (7.7)	6 (4.7)	0.298	1.68
Sleepiness	10 (5.2)	10 (11.0)	0.076	2.25	3 (4.6)	7 (5.5)	0.545	0.83
Weakened taste	9 (4.7)	11 (12.1)	**0.023**	**2.80**	0 (0.0)	9 (7.1)	**0.022**	--
Gingivitis, gum bleeding	8 (4.2)	6 (6.6)	0.273	1.62	2 (3.1)	6 (4.7)	0.453	0.64
Chest pain	5 (2.6)	5 (5.5)	0.186	2.17	0 (0.0)	5 (3.9)	0.123	--
Black tongue	4 (2.1)	6 (6.6)	0.062	3.32	0 (0.0)	4 (3.1)	0.188	--
Allergy	2 (1.0)	6 (6.6)	**0.015**	**6.71**	0 (0.0)	2 (1.6)	0.436	--
Nosebleed	2 (1.0)	1 (1.1)	0.689	1.06	0 (0.0)	2 (1.6)	0.436	--
Painful, swollen, and red tongue or mouth	1 (0.5)	6 (6.6)	**0.005**	**13.48**	0 (0.0)	1 (0.8)	0.661	--
Insomnia	1 (0.5)	4 (4.4)	**0.038**	**8.78**	0 (0.0)	1 (0.8)	0.661	--
Any adverse event,	76 (39.6)	48 (52.7)	**0.038**	**1.70**	29 (44.6)	47 (37.0)	0.308	1.37
mean (SD)	1.0 (1.8)	2.0 (2.7)	**0.005**	d = 0.39	0.9 (1.3)	1.1 (2.0)	0.563	d = 0.12
**Physiological Function**								
Breathing	67 (34.9)	12 (13.2)	**<0.001**	**0.28**	34 (52.3)	33 (26.0)	**<0.001**	**3.12**
Taste	54 (28.1)	12 (13.2)	**0.006**	**0.39**	26 (40.0)	28 (22.0)	**0.009**	**2.36**
Morning cough	54 (28.1)	12 (13.2)	**0.006**	**0.39**	24 (36.9)	30 (23.6)	0.052	1.89
Smell	51 (26.6)	10 (11.0)	**0.003**	**0.34**	24 (36.9)	27 (21.3)	**0.020**	**2.17**
Endurance	45 (23.4)	14 (15.4)	0.119	0.59	23 (35.4)	22 (17.3)	**0.005**	**2.61**
Physical status in general	43 (22.4)	14 (15.4)	0.170	0.63	24 (36.9)	19 (15.0)	**0.001**	**3.33**
Mood	41 (21.4)	8 (8.8)	**0.009**	**0.36**	20 (30.8)	21 (16.5)	**0.023**	**2.24**
Tooth discoloration	36 (18.8)	10 (11.0)	0.098	0.54	17 (26.2)	19 (15.0)	0.060	2.01
Stress tolerance	32 (16.7)	8 (8.8)	0.076	0.48	17 (26.2)	15 (11.8)	**0.012**	**2.64**
Quality of sleep	25 (13.0)	6 (6.6)	0.106	0.47	13 (20.0)	12 (9.4)	**0.040**	**2.40**
Sexual performance	21 (10.9)	4 (4.4)	0.070	0.37	11 (16.9)	10 (7.9)	0.057	2.38
Appetite	21 (10.9)	5 (5.5)	0.139	0.47	10 (15.4)	11 (8.7)	0.158	1.92
Memory	18 (9.4)	6 (6.6)	0.433	0.68	9 (13.8)	9 (7.1)	0.128	2.11
Craving to eat snacks	19 (9.9)	11 (12.1)	0.576	1.25	6 (9.2)	13 (10.2)	0.825	0.89
Any health improvement,	90 (46.9)	32 (35.2)	0.064	0.62	41 (63.1)	49 (38.6)	**0.001**	**2.72**
mean (SD)	2.7 (3.8)	1.5 (2.6)	**0.012**	d = 0.37	4.0 (4.2)	2.1 (3.4)	**<0.001**	**0.50**

Note: ^a^ Pearson chi-Square test or Mann–Whitney test *p*-values were used to examine the differences between current daily and former daily e-cigarette users. ^b^ Pearson chi-Square test or Fisher’s exact test or Mann–Whitney test *p*-values were used to examine the differences between exclusive e-cigarette users and dual users. ^c^ Reference: Current daily e-cigarette users. ^d^ Reference: Dual users. Bold values indicate statistically significant difference.

**Table 3:** Adverse events compared by nicotine-free and nicotine-containing e-liquid use among current daily users.

Adverse Events	OR	95% CI
Sore/dry mouth and throat	0.37	0.14–1.03
Cough	0.61	0.20–1.82
Burning, scratchy feeling in the mouth, lips, and throat	0.68	0.18–2.58
Headache	0.93	0.20–4.45
Palpitation	1.51	0.19–12.40
Dizziness	**0.20**	**0.04–0.90**
Breathing difficulties	0.94	0.11–8.04
Sleepiness	0.25	0.04–1.45
Weakened taste	**0.20**	**0.04–0.90**
Gingivitis, gum bleeding	**0.16**	**0.03–0.76**
Chest pain	**0.06**	**0.01–0.71**
Black tongue	**0.12**	**0.02–0.92**
Allergy	0.13	0.01–2.14
Nosebleed	0.13	0.01–2.14
Painful, swollen, and red tongue or mouth	N/A	N/A
Insomnia	N/A	N/A

Note: *n* = 169. Reference group: nicotine-free e-liquid. N/A: calculation of OR and 95% CI was not possible due to zero frequency in one cell. Bold values indicate statistically significant difference.

**Table 4:** Predictors of adverse events and perceived health improvement due to e-cigarette use among current daily users: Multiple binary logistic regression analyses.

Variables	Any Adverse Event ^a^		Any Perceived Health Improvement	
	OR	95% CI	OR	95% CI
**User Type**				
Exclusive EC user	1.02	0.46–2.22	**2.19**	**1.01** **–** **4.79**
Dual user	Ref.		Ref.	
**Sex**				
Female	0.62	0.26–1.49	0.75	0.32–1.73
Male	Ref.		Ref.	
**Age Group**				
31+ years old	0.39	0.15–1.00	1.27	0.52–3.11
18–30 years old	Ref		Ref.	
**Education Level**				
High school or higher education	1.55	0.65–3.70	0.60	0.26–01.38
Technical school or less	Ref.		Ref.	
**EC Device Type**				
Innovative Regulated Mod (IRM)	1.08	0.36–3.25	**4.36**	**1.37** **–** **13.84**
Advanced Personal Vaporizer (APV)	0.62	0.25–1.53	2.15	0.89–5.18
Cigalike or vape pen	Ref.		Ref.	
**Nicotine Content of the E-Liquid**				
Nicotine-containing	0.37	0.11–1.17	1.76	0.47–6.57
Nicotine free	Ref.		Ref.	
**Reason to Initiate Vaping**				
To quit smoking or avoid relapsing or smoking reduction	1.89	0.83–4.31	**2.82**	**1.24** **–** **6.39**
Other	Ref.		Ref.	
**Nagelkerke R^2^**	15.4%		22.7%	

Note: ^a^ *n* = 152. Ref. = Reference group. OR: odds ratio (adjusted). Bold values indicate statistically significant difference.

**Table 5:** Predictors of adverse events and perceived health improvement due to e-cigarette use among current and former daily users: multiple binary logistic regression analyses.

Variables	Any Adverse Event ^a^		Any Perceived Health Improvement	
	OR	95% CI	OR	95% CI
**User type**				
Former users	**2.27**	**1.17–4.39**	1.09	0.57–2.06
Current users	Ref.		Ref.	
**Sex**				
Female	1.38	0.71–2.69	1.49	0.79–2.83
Male	Ref.		Ref.	
**Age Group**				
31+ years old	**0.40**	**0.19–0.84**	0.68	0.34–1.37
18–30 years old	Ref.		Ref.	
**Education Level**				
High school or higher education	0.86	0.42–1.76	0.69	0.35–1.36
Technical school or less	Ref.		Ref.	
**EC Device Type**				
Innovative Regulated Mod (IRM)	1.43	0.59–3.45	**2.42**	**1.02–5.72**
Advanced Personal Vaporizer (APV)	0.77	0.38–1.55	1.29	0.66–2.53
Cigalike or vape pen	Ref.		Ref.	
**Nicotine Content of the E-Liquid**				
Nicotine-containing	**0.29**	**0.11–0.75**	1.12	0.46–2.77
Nicotine-free	Ref.		Ref.	
**Reason to Initiate Vaping**				
To quit smoking or avoid relapsing or smoking reduction	**2.30**	**1.17–4.39**	**2.62**	**1.37–5.00**
Other	Ref.		Ref.	
**Nagelkerke R^2^**	17.8%		11.4%	

Note: ^a^ *n* = 209. Ref. = Reference group. OR: odds ratio (adjusted). Bold values indicate statistically significant difference.

## Data Availability

The data used in this study are available on request from the corresponding author.
